# Systematic review of the perioperative immunotherapy in patients with non-small cell lung cancer: evidence mapping and synthesis

**DOI:** 10.3389/fonc.2023.1092663

**Published:** 2023-04-27

**Authors:** Yunfeng Ni, Jie Lei, Wan Huang, Jian Wang, Haihua Guo, Feng Lv, Shuhong Kang, Ke Lan, Tao Jiang

**Affiliations:** ^1^ Department of Thoracic Surgery, Tangdu Hospital, Air Force Military Medical University, Xi’an, China; ^2^ Department of Cell Biology, National Translational Science Center for Molecular Medicine, Fourth Military Medical University, Xi’an, China

**Keywords:** perioperative immunotherapy for NSCLC immunotherapy, neoadjuvant therapy, adjuvant therapy, non-small cell lung cancer, evidence mapping, scoping review

## Abstract

**Objectives:**

This study aimed to use evidence mapping to provide an overview of immune checkpoint inhibitors (ICIs) as perioperative treatments for non-small cell lung cancer (NSCLC) and to identify areas of this field where future research is most urgently needed.

**Methods:**

Multiple databases (PubMed, EMBASE, Cochrane Library, and Web of Science) were searched to identify clinical trials published up to November 2021 that examined the effect of perioperative ICIs for perioperative treatment of NSCLC. Study design, sample size, patient characteristics, therapeutic regimens, clinical stages, short-term and long-term therapeutic outcomes, surgery associated parameters, and therapeutic safety were examined.

**Results:**

We included 66 trials (3564 patients) and used evidence mapping to characterize the available data. For surgery associated outcomes, sixty-two studies (2480 patients) provided complete information regarding the use of surgery after neoadjuvant immunotherapy and data on R0 resection were available in 42 studies (1680 patients); for short-term clinical outcomes, 57 studies (1842 patients) evaluated pathologic complete response (pCR) after neoadjuvant immunotherapy and most of included studies achieved pCR in the range of 30 to 40%; for long-term clinical outcomes, 15 studies (1932 patients) reported DFS, with a median range of 17.9-53.6 months; and only a few studies reported the safety profiles of perioperative immunotherapies.

**Conclusion:**

Our evidence mapping systematically summarized the results of all clinical trials and studies that examined ICIs as perioperative treatments for NSCLC. The results indicated more studies that evaluate long-term patient outcomes are needed to provide a stronger foundation for the use of these treatments.

## Introduction

Lung cancer is the leading cause of cancer deaths and has a high prevalence and mortality worldwide (1). Non-small cell lung cancer (NSCLC) is the most common type of lung cancer, accounting for 85% of malignancies (2). Adenocarcinoma and squamous cell carcinoma are the two most common pathological subtypes of NSCLC (2). Surgical resection remains the preferable treatment for operable NSCLC. However, the probability of cure remains low due to the high recurrence rate after surgery, especially in patients diagnosed with late-stage NSCLC (3). Although chemotherapy can only increase the 5-year survival rate of NSCLC by 4 to 8% after surgery, it is still the primary choice among the many options for perioperative treatment (4–7). In addition, the rate of post-surgery recurrence remains high (20–30% for stage I, 50% for stage II, and 60% for stage IIIa) (6). Furthermore, it was reported that there was no evidence of a difference in the prognosis of patients with resectable lung cancer between the neoadjuvant and adjuvant chemotherapy (8). Therefore, the benefit of conventional chemotherapy as a neoadjuvant or adjuvant treatment is unsatisfactory. On the contrary, because of recent advances in immunotherapy for NSCLC, immune checkpoint inhibitors (ICIs) have become a promising alternative for the perioperative treatment of NSCLC (9–11).

Immunotherapies that target immune suppressive checkpoints, such as cytotoxic lymphocyte antigen 4 (CTLA-4), programmed cell death 1 (PD-1), and the PD-1 ligand (PD-L1), have changed the treatment paradigm used for many malignant diseases, especially NSCLC (12–14). Immunotherapy was first applied to NSCLC as a systemic treatment for inoperable tumors, and the results indicated it provided a durable response as an upfront treatment and for relapsed disease (15, 16). Then, many recent clinical trials have assessed the value of the immunotherapies as perioperative treatments for operable NSCLC and demonstrated that perioperative immunotherapies decreased tumor stage, enhanced the rate of complete resection, and reduced recurrence after surgery (17). Although numerous clinical studies examined different ICIs as single agents or combined with conventional chemotherapy/radiotherapy as perioperative treatments for NSCLC, and these studies differed in design, population characteristics, therapeutic regimens, and treatment times (18–22). There are many ongoing clinical trials examining the evidence gap regarding the efficacy and safety of ICIs as perioperative treatments for NSCLC (23–26). However, it is necessary to thoroughly understand the currently available data on this topic.

Evidence mapping is an emerging method that provides a comprehensive visual presentation of all available evidence on various topics (27, 28). It provides a clear and straightforward summary and visual presentation of pooled data. In addition, it is more flexible than meta-analysis because it analyzes data of different studies that cannot be used for a pooled statistical analysis (27). To obtain an in-depth understanding of currently available data regarding the efficacy and safety of ICIs as perioperative treatments for NSCLC, we systematically reviewed all relevant studies and presented the results using evidence mapping. In addition, we applied for a tabular review for specific parameters whose data were available in a small number of studies. This review aims to identify data gaps in our current understanding of perioperative immunotherapy for NSCLC and help guide future studies on this topic.

## Methods

### Literature search

We searched PubMed, EMBASE, Cochrane Library, and the Web of Science until July 08, 2022, to identify registered trials that examined perioperative immunotherapy for NSCLC that were published before the search date, without limitations on date or time but restriction to the English language. Advanced search functions of each database were used to search the following terms: (“non-small cell lung cancer” or “lung adenocarcinoma” or “squamous cell lung cancer”) and (“immunotherapy” or “immune checkpoint inhibitor” or “programmed death ligand 1” or “programmed death 1” or “cytotoxic lymphocyte antigen 4” or “nivolumab” or “pembrolizumab” or “durvalumab” or “atezolizumab” or “ipililumab” or “camrelizumab” or “sintilimab” or “toripalimab”) and (“perioperative” or “adjuvant” or “neoadjuvant”). Two investigators performed the search independently and worked together to identify eligible studies before further analysis. Some important international conferences’ websites (e.g., ESMO, WCLC, ASCO, AACR, and ELCC) were searched for more eligible papers. We performed this present review following a project outline without published protocol or registration. We reported the study following the Preferred Reporting Items for Systematic Reviews and Meta-Analyses (PRISMA) extension for scoping reviews (29).

### Inclusion and exclusion criteria

A registered trial that examined NSCLC that met the following three general inclusion criteria was considered eligible. First, the study was an interventional (clinical trial). Second, the study used any single immunotherapy agent or a combination of any immunotherapy agent with any other drug during the perioperative period, including neoadjuvant and/or adjuvant treatment regimens. The immunotherapy agents included (but were not limited to) inhibitors of PD-1, PD-L1, CTLA-4, T cell immunoreceptor with immunoglobulin and ITIM domain (TIGIT), lymphocyte activation gene 3 (LAG-3), and T cell immunoglobulin-3 (TIM-3). There was no limitation on dosage, treatment frequency, regimen, treatment duration, use of other combined therapy, or lines of treatment. Third, at least one of the following outcome parameters was evaluated: pathologic complete response (pCR), major pathologic response (MPR), overall survival (OS), disease-free survival (DFS), progression-free survival (PFS), recurrence-free survival (RFS), event-free survival (EFS), surgery outcomes, and safety outcomes.

Studies were excluded if the study population overlapped with another study, if there were no eligible data available for extraction, or if it was written in a non-English language. A PRISMA flow diagram was used to examine the full study-selection process (29).

### Data collection

Data from each study were extracted by one author and double-checked by another author using a standardized data extraction form. Any disagreement was resolved by discussion, with assistance from a third author if necessary. If information relating to a potentially eligible study was lacking, the study authors were contacted to request this information. A Population, Intervention, Comparison, and Outcomes (PICOS) structure was used to formulate the data extraction according to general study characteristics (first author’s name, year of publication, country, name of research center, funding sources), participants (diagnosis, diagnostic criteria, clinical-stage, inclusion criteria, exclusion criteria, sample size, patient gender and age, disease stage, smoking history, Eastern Cooperative Oncology Group performance score, TNM stage, PD-L1 status, tumor mutational burden (TMB), and comorbidities), interventions (treatment frequency, dosage, treatment duration, type of perioperative approach), outcomes (types of outcomes, definitions, measurement times); Results (all relevant dichotomous and continuous results); and study design (randomized controlled trial (RCT), non-randomized controlled study or single-arm study).

### Data summary and visualization

Evidence mapping was utilized to summarize the following basic characteristic of the included trials: study design (single-arm, non-randomized controlled study, or RCT), perioperative subtype (neoadjuvant, adjuvant, or both), and clinical cancer stage(s) of the study population. The association of variance in surgical parameters, therapeutic outcome, and safety profile with perioperative treatment in the different studies was also summarized and presented using evidence mapping. A tabular review was used to summarize certain parameters whose information was only available for a limited number of studies or in a format inconvenient for evidence mapping.

#### Bubble plot of the basic characteristics of studies

One bubble plot was used to summarize the basic characteristics of the included studies. In this plot, each bubble represented a group of studies with the same design, patients with the same clinical stage, and patients receiving the same perioperative therapy. This information was displayed in the plot as follows: (*i*) the horizontal axis indicated the type of perioperative therapy as “Neoadjuvant”, “Adjuvant”, “Neoadjuvant + Adjuvant”, and “Neoadjuvant ± Adjuvant”; (*ii*) the vertical axis indicated the clinical stage as “Stage I to II”, “Stage I to III”, “Stage I to IV”, “Stage II to III,” “Stage II to IV”, “Stage III only,” and “Stage III to IV”; (*iii*) bubble color indicated study design, in which red indicated “single arm study”, yellow indicated “non-randomized controlled study”, and green indicated “RCT”; (*iv*) bubble size indicated the number of studies in each group; and (*v*) bubble label indicated the number of studies in each group.

#### Bubble plots of study outcomes

Five other bubble plots were used to examine dichotomous outcomes when there were more than ten results. In these plots, each bubble represented a group of studies with the same design, patients with the same clinical stage, and patients receiving the same neoadjuvant intervention. This information was displayed in each plot as follows: (*i*) the horizontal axis indicated clinical stage as “Stage I to II,” “Stage I to III,” “Stage I to IV”, “Stage II to III,” “Stage II to IV”, “Stage III only,” and “Stage III to IV”; (*ii*) the vertical axis indicated the incidence rate (%) of the outcome for each group as “Surgery Rate,” “R0 Resection”, “Delay of Surgery Rate,” “pCR,” and “MCR” (obtained using a single-arm meta-analysis with the R package ‘meta’ and a random-effects model for data synthesis due to the variety of clinical stages and intervention types); (*iii*) bubble color indicated study design; (*iv*) bubble size indicated the total number of patients; and (*v*) bubble label indicated the type of neoadjuvant intervention the total number of patients.

## Results

### Study selection

**Figure 1:**
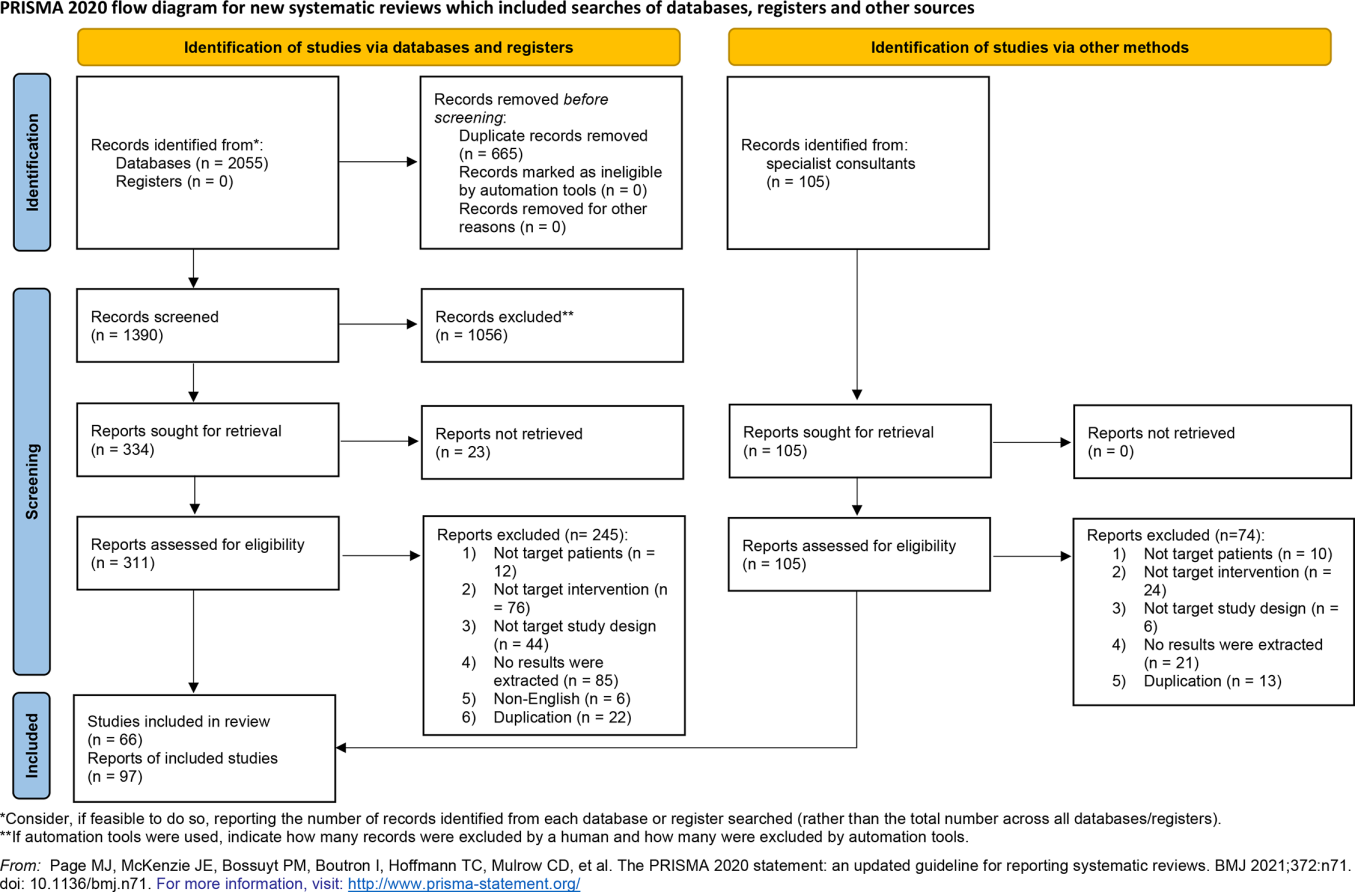
PRISMA flow diagram of the methods used to identify, screen, and include eligible studies that examined perioperative immunotherapy in patients with non-small cell lung cancer.

We identified 2160 studies from the primary search, including 2055 studies from public databases. After the removal of 665 duplicate references, we screened 1390 records. We also identified 105 records using other methods and screened them for eligibility. After thorough screening for eligibility based on pre-defined inclusion and exclusion criteria, we identified 66 eligible studies with 97 reports for analysis (**Figure 1**).

#### Basic characteristics of included studies

**Figure 2:**
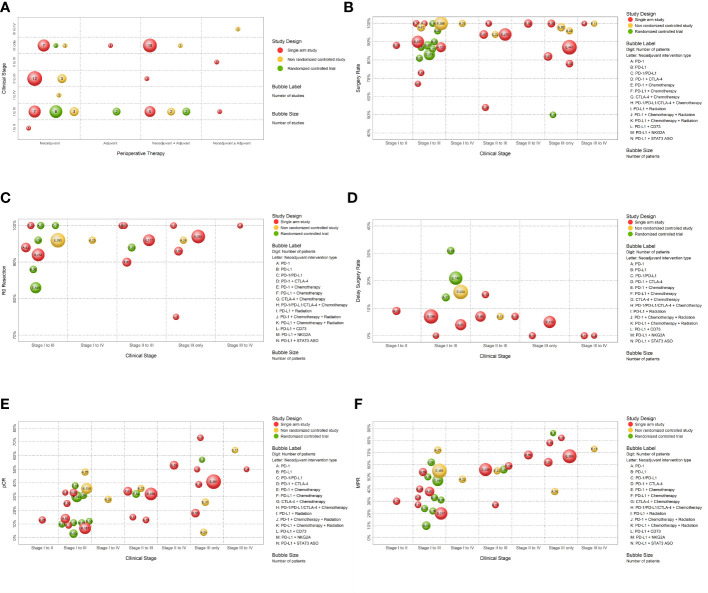
The evidence maps. **(A)** The evidence map of the basic characteristics of included studies. **(B)** The evidence map of the surgery rate according to therapeutic intervention and study design. **(C)** The evidence map of the rate of R0 resection according to therapeutic intervention and study design. **(D)** The evidence map of the rate of surgery delay according to therapeutic intervention and study design. **(E)** The evidence map of pathological complete response according to therapeutic intervention and study design. **(F)** The evidence map of major pathologic response according to therapeutic intervention and study design.

We initially used evidence mapping to describe the basic characteristics of all 66 included studies (**Table 1**; **Figure 2A**). Seven were RCTs, 13 were non-randomized controlled studies, and 46 were single arm studies. Analysis of perioperative subtypes indicated that 44 studies used neoadjuvant treatment alone, 3 used adjuvant treatment alone, 21 used neoadjuvant plus adjuvant treatment, and the remaining 3 used neoadjuvant therapy with or without adjuvant treatment without adjuvant therapy based on the physician’s personal decision. The sample size of the included studies varied from 4 to 590 patients, and there were 3564 patients in total. Among all included NSCLC patients, 22.52% had lung adenocarcinoma, 27.89% had lung squamous cell carcinoma, and explicit pathological information was not provided for the other 49.41%. 53.7% of the patients had stage III cancer (according to AJCC 7/8, or if the staging guideline was not mentioned), and patients with stage I cancer only accounted for 1% of all cases. Thus, most of the included studies examined patients with stage II to III NSCLC.

**Table 1:** Detailed information of all included studies.

Perioperative period	Clinical stage	Intervention	Study ID	Study design	Immune checkpoint inhibitor	Other regimen	Phase	Stage	Sample size*
Neoadjuvant	Stage I to II	Neoadjuvant: PD-1	MK3475-223Bar 2019(NCT02938624)	Single arm study	Neoadjuvant: Pembrolizumab (200 mg) Q3W x 1-2 cycles	–	I	I-II	26
	Stage I to III	Neoadjuvant: PD-1	Zhao 2021	Nonrandomized controlled study	Arm 1: Neoadjuvant: Pembrolizumab (200mg)Arm 2: Neoadjuvant: No ICIArm 3: Neoadjuvant: No ICI	Arm 1: Neoadjuvant: N/AArm 2: Neoadjuvant: EGFR-TKIArm 3: Neoadjuvant: Chemotherapy	Retrospective study	IB-IIIB	25 (177)
			NEOSTARCascone 2021 arm1(NCT03158129)	Randomized controlled trial	Arm 1: Neoadjuvant: Nivolumab (3 mg/kg) Q2W x 3 cyclesArm 2: Neoadjuvant: Nivolumab (3 mg/kg) Q2W x 3 cycles + Ipilimumab (1 mg/kg) Q6Wx 1 cycle	–	II	IA–IIIA (N2 single station) (AJCC v7)	23 (44)
			CheckMate159Bott 2019(NCT02259621)	Single arm study	Neoadjuvant: Nivolumab (3 mg/kg) Q2W x 2 cycles	–	Ib/II	I –IIIA	21
		Neoadjuvant: PD-L1	IFCT-1601 IONESCOWislez 2020(NCT03030131)	Single arm study	Neoadjuvant: Durvalumab (750 mg) Q2W x 3 cycles	–	II	IB > 4cm-IIIA (non N2) (AJCC v8)	46
			PRINCEPSBesse 2020(NCT02994576)	Single arm study	Neoadjuvant: Atezolizumab (1200 mg) Q3W x 1 cycle	–	II	IA (≥2cm)-IIIA(non N2)	30
		Neoadjuvant: PD-1 + Chemotherapy	Hu 2021	Single arm study	Neoadjuvant: Pembrolizumab/Tislelizumab/Sintilimab/Toripalimab x 2-4 cycles	Neoadjuvant: Carboplatin + Pemetrexed/Carboplatin+ Paclitaxel/Cisplatin + Gemcitabine/Cisplatin + Pemetrexed/Cisplatin + Paclitaxel x 2-4 cycles	IIT	Ib–IIIb	20
			CheckMate816Forde 2021 arm1(NCT02998528)	Randomized controlled trial	Arm 1: Neoadjuvant: Nivolumab (360 mg) Q3W x 3 cycles Arm 2: Neoadjuvant: No ICI Arm 3: Neoadjuvant: Nivolumab (3 mg/kg) Q2W x 3 cycles + Ipilimumab (1 mg/kg) Q6W x 1 cycle	Arm 1: Neoadjuvant: SQ: Gemcitabine + Cisplatin/Paclitaxel + Carboplatin Q3W x 3 cycles, NSQ: Pemetrexed + Cisplatin/Paclitaxel + Carboplatin Q3W x 3 cycles Arm 2: Neoadjuvant: SQ: Vinorelbine + Cisplatin/Docetaxel + Cisplatin/Gemcitabine + Cisplatin/Paclitaxel + Carboplatin Q3W x 3 cycles, NSQ: Pemetrexed + Cisplatin/Paclitaxel + Carboplatin Q3W x 3 cycles Arm 3: Neoadjuvant: N/A	III	IB (≥4 cm)–IIIA (per AJCC 7th ed)	179 (469)
		Neoadjuvant: PD-L1 + Chemotherapy	Tfayli 2020 (NCT03480230)	Single arm study	Neoadjuvant: Avelumab (10 mg/kg) Q2W x 4 cycles	Neoadjuvant: SQ: Cisplatin (75 mg/m^2^, D1)/Carboplatin (AUC 5, D1) + Gemcitabine (1000 mg/m^2^), D1,8] Q2W x 4 cycles; NSQ: Cisplatin (75 mg/m^2^, D1)/Carboplatin (AUC 5, D1) + Pemetrexed (500 mg/m^2^, D1) Q2W x 4 cycles	II	IB (>4 cm in size) -IIIA (AJCC v8)	15
		Neoadjuvant: PD-1 + CTLA-4	NEOSTARCascone 2021 arm2(NCT03158129)	Randomized controlled trial	Arm 1: Neoadjuvant: Nivolumab (3 mg/kg) Q2W x 3 cyclesArm 2: Neoadjuvant: Nivolumab (3 mg/kg) Q2W x 3 cycles + Ipilimumab (1 mg/kg) Q6W x 1 cycle	–	II	IA–IIIA (N2 single station) (AJCC v7)	21 (44)
			Reuss 2020(NCT02259621)	Single arm study	Neoadjuvant: Nivolumab (3 mg/kg) Q2W x 3 cycles + Ipilimumab (1 mg/kg) Q6W x 1 cycle	–	Ib/II	IB (≥4 cm)–IIIA	9
			CheckMate816Forde 2021 arm2(NCT02998528)	Randomized controlled trial	Arm 1: Neoadjuvant: Nivolumab (360 mg) Q3W x 3 cyclesArm 2: Neoadjuvant: No ICIArm 3: Neoadjuvant: Nivolumab (3 mg/kg) Q2W x 3 cycles + Ipilimumab (1 mg/kg) Q6Wx 1 cycle	Arm 1: Neoadjuvant: SQ: Gemcitabine + Cisplatin/Paclitaxel + Carboplatin Q3W x 3 cycles, NSQ: Pemetrexed + Cisplatin/Paclitaxel + Carboplatin Q3W x 3 cyclesArm 2: Neoadjuvant: SQ: Vinorelbine + Cisplatin/Docetaxel + Cisplatin/Gemcitabine + Cisplatin/Paclitaxel + Carboplatin Q3W x 3 cycles, NSQ: Pemetrexed + Cisplatin/Paclitaxel + Carboplatin Q3W x 3 cyclesArm 3: Neoadjuvant: N/A	III	IB (≥4 cm)–IIIA (per AJCC 7th ed)	111 (469)
	Stage II to III	Neoadjuvant: PD-1	NEOMUNEichhorn 2021(NCT03197467)	Single arm study	Neoadjuvant: Pembrolizumab (200 mg) Q3W x 2 cycles	–	II	II/IIIA(AJCC v7)	15
		Neoadjuvant: PD-1 + Chemotherapy	Chen 2020a	Non randomized controlled study	Arm 1: Neoadjuvant: Pembrolizumab (100 mg) Q3W x 2 cyclesArm 2: Neoadjuvant: No ICI	Arm 1: Neoadjuvant: Paclitaxel (135 mg/m^2^) + Cisplatin (75 mg/m^2^) Q3W x 2 cyclesArm 2: Neoadjuvant: Paclitaxel (135 mg/m^2^) + Cisplatin (75 mg/m^2^) Q3W x 2 cycles	–	IIB–IIIA	4 (9)
			Liang 2021	Non randomized controlled study	Arm 1: Neoadjuvant: PD-1 Q3W x not fixed cyclesArm 2: Neoadjuvant: No ICI	Arm 1: Neoadjuvant: Chemotherapy Q3W x not fixed cyclesArm 2: Neoadjuvant: Chemotherapy Q3W x not fixed cycles	Retrospective study	Unresectable due to bulky N2, multi-station N2 or invasion of critical structures	10 (20)
			Hong 2021	Single arm study	Neoadjuvant: Pembrolizumab/Sintilimab/Camrelizumab x 2-4 cycles	Neoadjuvant: Paclitaxel + Cisplatin (Carboplatin) x 2-4 cycles	Retrospective study	IIA–IIIC	25
			Shen 2021	Single arm study	Neoadjuvant: Pembrolizumab (2 mg/kg) Q3W x 2 cycles	Neoadjuvant: Nab-paclitaxel (100 mg/m^2^, D1, 8) + Carboplatin (AUC 5) Q3W x 2 cycles	II	IIB–IIIB (/AJCC v7)	37
			Jiang 2021	Single arm study	Neoadjuvant: Pembrolizumab/Nivolumab	Neoadjuvant: ± Chemotherapy	Retrospective study	IIA–IIIB	31
			Duan 2021	Single arm study	Neoadjuvant: Nivolumab (360/200 mg)/Pembrolizumab (200 mg)/Sintilimab (200 mg) x 3-4 cycles	Neoadjuvant: Chemotherapy x 3-4 cycles	–	IIA–IIIB	23
		Neoadjuvant: PD-L1 + Chemotherapy	Shu 2020(NCT02716038)	Single arm study	Neoadjuvant: Atezolizumab (1200 mg) Q3W x 4 cycles	Neoadjuvant: Nab-paclitaxel (100 mg/m^2^, D1,8,15) + Carboplatin (AUC 5, D1) Q3W x 4 cycles	II	IB–IIIA (AJCC v7)	30
		Neoadjuvant: CTLA-4 + Chemotherapy	Boyer 2016(NCT01820754)	Single arm study	Neoadjuvant: Paclitaxel (175 mg/m^2^) + Cisplatin (75 mg/m^2^)/Carboplatin (AUC 6) Q3W x 1 cycle → Ipilimumab (10 mg/kg) + chemotherapy (as in cycle 1) Q3W x 2 cycles;Adjuvant: Radiation		II	IB-IIIA	9
	Stage III only	Neoadjuvant: PD-1	Huang 2021	Non randomized controlled study	Arm 1: Neoadjuvant: Nivolumab (3 mg/kg) Q3W x 2 cycles;Arm 2: Neoadjuvant: No ICI	Arm 1: Neoadjuvant: N/AArm 2: Neoadjuvant: Gemcitabine (1000 mg/m2, D1,8) + Cisplatin (80 mg/m^2^, D1) Q3W x 2 cycles	Retrospective study	IIIA (N2)	25 (107)
		Neoadjuvant: PD-1 + Chemotherapy	Chen 2021b	Single arm study	Neoadjuvant: Pembrolizumab (2 mg/kg) Q3W x 2 cycles	Neoadjuvant: SQ: Cisplatin (75 mg/m^2^) + Paclitaxel liposome 135 mg/m^2^ Q3W x 2 cycles; NSQ: Cisplatin (75 mg/m^2^) + Pemetrexed (500 mg/m^2^) Q3W x 2 cycles	–	–	35
			Lei 2020(NCT04338620)	Randomized controlled trial	Arm 1: Neoadjuvant: Camrelizumab (200 mg) Q3W x 3 cyclesArm 2: Neoadjuvant: No ICI	Arm 1: Neoadjuvant: Nab-paclitaxel (130 mg/m^2,^D1,8) + Cisplatin (75 mg/m^2^, D1) Q3W x 3 cyclesArm 2: Neoadjuvant: Nab-paclitaxel (130 mg/m^2^, D1,8) + Cisplatin (75 mg/m^2^, D1) Q3W x 3 cycles	II	IIIA-IIIB (N2)	14 (27)
			Zhang 2021(NCT04324151)	Single arm study	Neoadjuvant: Toripalimab/Pembrolizumab x 2 cycles	Neoadjuvant: Chemotherapy x 2 cycles	Retrospective study	IIIA-IIIB	30
			Wang 2021	Single arm study	Neoadjuvant: Nivolumab (200mg)/Pembrolizumab (100mg)/Camrelizumab(200mg) Q3W x 2 cycles	Neoadjuvant: Nab-paclitaxel (100 mg/m^2^, D1,8)+ Carboplatin (AUC 5, D1) Q3W x 2 cycles	Prospective study	IIIA	72
	Stage III to IV	Neoadjuvant: PD-1/PD-L1	Walles 2020	Single arm study	Neoadjuvant: Pembrolizumab/Atezolizumab x ≥2 cycles	–	Retrospective study	III- IV	4
Neoadjuvant + Adjuvant	Stage I to III	Neoadjuvant: PD-1;Adjuvant: ± Chemotherapy → PD-1	Ready 2019	Single arm study	Neoadjuvant: Pembrolizumab (200mg) Q3W x 2 cycles;Adjuvant: ± Chemotherapy → Pembrolizumab (200mg) Q3W x 4 cycles		–	IB-IIIA	30
		Neoadjuvant: PD-1;Adjuvant: PD-1 ± Chemotherapy ± Radiotherapy	TOP1501Tong 2021(NCT02818920)	Single arm study	Neoadjuvant: Pembrolizumab (200 mg) Q3W x 2 cycles;Adjuvant: Pembrolizumab (200 mg) Q3W x 4 cycles	Adjuvant: ± Chemotherapy ± Radiation	II	IB-IIIA	30
		Neoadjuvant: CTLA-4 + Chemotherapy; Adjuvant: CTLA-4	TOP1201Yi 2017(NCT01820754)	Single arm study	Neoadjuvant: Paclitaxel (175 mg/m^2^) + Cisplatin (75 mg/m^2^)/Carboplatin (AUC 6) Q3W x 1 cycle → Ipilimumab (10 mg/kg) + Chemotherapy (as in cycle 1) Q3W x 2 cycles;Adjuvant: Ipilimumab (10 mg/kg) Q3W x 2 cycles → Ipilimumab (10 mg/kg) Q12W x 2 cycles		II	IB-IIIA	24
	Stage III only	Neoadjuvant: PD-1 + Chemotherapy;Adjuvant: PD-1 ± Chemotherapy	Chen 2021c	Single arm study	Neoadjuvant: Pembrolizumab x 4 cycles/Nivolumab x 2 cycles;Adjuvant: Pembrolizumab/Nivolumab x 12 months	Neoadjuvant: Carboplatin + Paclitaxel x 4 cycles;Adjuvant: ± Carboplatin + Paclitaxel x 12 months	Retrospective study	IIIA-IIIB(AJCC v8)	12
		Neoadjuvant: PD-1 + Chemotherapy; Adjuvant: ± Radiotherapy + PD-1	NADIMProvencio 2020(NCT03081689)	Single arm study	Neoadjuvant: Nivolumab (360 mg) Q3W x 3 cycles;Adjuvant: Nivolumab (240 mg) Q2W x 4 months →Nivolumab (480 mg) Q4W x 8 months	Neoadjuvant: Paclitaxel (200 mg/m^2^, D1) + Carboplatin (AUC 6, D1) Q3W x 3 cycles	–	IIIA	46
		Neoadjuvant: PD-1 + Chemotherapy + Radiotherapy;Adjuvant: PD-1	Lemmon 2020(NCT02987998)	Single arm study	Neoadjuvant: Pembrolizumab (200mg) Q3W x 3 cycles;Adjuvant: Pembrolizumab (200mg) Q3W x 6 months	Neoadjuvant: Cisplatin (50mg/m^2^, D1,8,29,36) + Etoposide (50mg/m^2^, D1-5, 29-35) + Radiotherapy (45Gy in 25 Fx)	I	IIIA	9
		Neoadjuvant: PD-L1 + Chemotherapy; Adjuvant: ± Radiotherapy + PD-L1	SAKK 16/14Rothschild 2020(NCT02572843)	Single arm study	Neoadjuvant: Cisplatin (100 mg/m^2^) + Docetaxel (85 mg/m^2^) Q3W x 3 cycles → Durvalumab (750 mg) Q2W x 2 cycles;Adjuvant: ± Radiotherapy + Durvalumab (750 mg) Q2W x 12 months		II	IIIA (N2) (AJCC v7)	67
		Neoadjuvant: PD-L1 + Chemotherapy + Radiotherapy;Adjuvant: PD-L1	ACTS-30Hong 2021(NCT03694236)	Single arm study	Neoadjuvant: Durvalumab (1500mg) Q4W x 2 cycles;Adjuvant: Durvalumab (1500mg) Q4W x 12 months	Neoadjuvant: Paclitaxel (45mg/m2) + Carboplatin (AUC 2) Q1W x 5 cycles+ Radiotherapy (45Gy in 25 Fx)	–	III	24
Neoadjuvant ± Adjuvant	Stage I to III	Neoadjuvant: PD-1;Adjuvant: ± PD-1/PD-1+ Chemotherapy/Chemotherapy ± Radiatherapy	Gao 2020(ChiCTR-OIC-17013726)	Single arm study	Neoadjuvant: Sintilimab (200 mg) Q3W x 2 cycles;Adjuvant: ± (Chemotherapy+Sintilimab/Chemotherapy ± Radiatherapy/Sintilimab)	–	–	IA–IIIB	40
		Neoadjuvant: PD-L1;Adjuvant: ± PD-L1/stage-appropriate therapy	LCMC3Lee 2020(NCT02927301)	Single arm study	Neoadjuvant: Atezolizumab (1200 mg) Q3W x 2 cycles;Adjuvant: ± Atezolizumab (1200 mg) Q3W x 12 months	Adjuvant: ± Stage-appropriate therapy	II	IB-IIIB	181
		Neoadjuvant: PD-L1Adjuvant: ± PD-L1/Chemotherapy/Radiatherapy/Chemotherapy+Radiatherapy	Altorki 2021 arm1(NCT02904954)	Randomized controlled trial	Arm 1: Neoadjuvant: Durvalumab (1.12 g) Q3W x 2 cycles;Adjuvant: ± Durvalumab (1.12 g) Q4W x 12 cyclesArm 2: Neoadjuvant: Durvalumab (1.12 g) Q3W x 2 cycles;Adjuvant: ± Durvalumab (1.12 g) Q4W x 12 cycles	Arm 1: Neoadjuvant: N/A; Adjuvant: Adjuvant: ± Chemotherapy/Radiatherapy/Chemotherapy+/RadiatherapyArm 2: Neoadjuvant: SBRT (24Gy in 3 Fx); Adjuvant: ± Chemotherapy/Radiatherapy/Chemotherapy+Radiatherapy	II	I/IIIA (AJCC v7)	30 (60)
		Neoadjuvant: PD-L1 + RadiatherapyAdjuvant: ± PD-L1/Chemotherapy/Radiatherapy/Chemotherapy+Radiatherapy	Altorki 2021 arm2(NCT02904954)	Randomized controlled trial	Arm 1: Neoadjuvant: Durvalumab (1.12 g) Q3W x 2 cycles;Adjuvant: ± Durvalumab (1.12 g) Q4W x 12 cyclesArm 2: Neoadjuvant: Durvalumab (1.12 g) Q3W x 2 cycles;Adjuvant: ± Durvalumab (1.12 g) Q4W x 12 cycles	Arm 1: Neoadjuvant: N/A; Adjuvant: Adjuvant: ± Chemotherapy/Radiatherapy/Chemotherapy+/RadiatherapyArm 2: Neoadjuvant: SBRT (24Gy in 3 Fx); Adjuvant: ± Chemotherapy/Radiatherapy/Chemotherapy+/Radiatherapy	II	I/IIIA (AJCC v7)	30 (60)
	Stage III only	Neoadjuvant: PD-1 + Chemotherapy;Adjuvant: ± PD-1	NeoTAP01Zhao 2021(NCT04304248)	Single arm study	Neoadjuvant: Toripalimab (240 mg) Q3W x 3 cycles;Adjuvant: ± Toripalimab (240 mg) Q3W x 12 months	Neoadjuvant: Carboplatin (AUC 5, D1) + Pemetrexed (500 mg/m^2^, D1) Q3W x 3 cycles; Other subtypes: Carboplatin (AUC 5, D1) + Nab-paclitaxel (260 mg/m^2^, D1) Q3W x 3 cycles	–	IIIA-IIIB (T3N2)	33
		Neoadjuvant: PD-1 + Chemotherapy;Adjuvant: ± Chemotherapy/PD-1/ICI + Chemotherapy	Deng 2021	Single arm study	Neoadjuvant: Pembrolizumab/Nivolumab/Sintilimab/Tislelizumab/Camrelizumab x ≥ 2 cycles	Neoadjuvant: Chemotherapy;Adjuvant: ± Chemotherapy/PD-1/ICI + Chemotherapy	Retrospective study	IIIB	51
Adjuvant	Stage I to III	Arm 1: Adjuvant: Chemotherapy→PD-L1Arm 2: Chemotherapy	IMpower 010Wakelee 2021(NCT02486718)	Randomized controlled trial	Arm 1: Chemotherapy Q3W x 4 cycles →Atezolizumab (1200 mg) Q3W x 16 cyclesArm 2: Chemotherapy Q3W x 4 cycles		III	IB-IIIA (AJCC v7)	507 (1005)
	Stage III only	Adjuvant: PD-1	Park 2019(NCT03053856)	Single arm study	Adjuvant: Pembrolizumab (200 mg) Q3W x 2 years	–	II	IIIA (N2)	37

*: The number in parentheses is the overall sample size included, and the number in front of the parentheses is the sample size of the ICI group.

AC, Adenocarcinoma; SBRT, Stereotactic body radiotherapy; SQ, Squamous cell cancer; NSQ, Non-squamous cell cancer.

### Surgery associated outcomes

We systematically summarized the parameters associated with surgical procedure and surgical outcome to examine the relationship between neoadjuvant immunotherapy with surgery (**sTable 1**). The studies commonly used surgery rate, complete resection rate, downstaging rate, and surgery delay rate to assess the direct impact of neoadjuvant immunotherapy on surgical treatment. R0 resection (complete resection) was the most important surgical outcome used to assess the therapeutic efficacy of neoadjuvant immunotherapy.

### Surgery

Disease progression and other complications during neoadjuvant therapy might affect the use of surgery. Sixty-two studies (2480 patients) provided complete information regarding the use of surgery after neoadjuvant immunotherapy, including 5 RCTs, and 12 non-randomized controlled studies and 44 single-arm studies, (**sTable 1**). Thirty studies reported a surgery rate of 100% and 53 reported a surgery rate of at least 80% (**Figure 2B**). Only one RCT had a surgery rate below 80%, but this study had a small sample size (**Figure 2B**; **sTable 1**).

### R0 resection

Data on R0 resection were available in 42 studies (1680 patients), including 3 RCTs, 6 non-randomized controlled studies, and 33 single-arm studies (**sTable 1**). Most of these patients had clinical stage I to III NSCLC. Twelve studies used a mono-immunotherapy as neoadjuvant therapy, one used a dual ICIs, and 31 used a combination of ICI and chemotherapy and/or radiotherapy. A total 20 studies reported R0 resection rates of 100%, 40 reported R0 resection rates above 80%, and the remaining two reported R0 resection rates of 75.0% and 77.4% (**Figure 2C**). The study with the lowest R0 (75.0%) only examined eight patients who had stage IIIA NSCLC.

### Surgery delay

Various factors, such as adverse effects of neoadjuvant treatment and disease progression, could delay surgery. There were 25 studies provided complete information regarding surgery delay and 12 studies reported no surgery delay (**Figure 2D**).

### Minimally invasive surgery

We summarized other surgery-associated parameters in a tabular format because the data were insufficient for evidence mapping. 35 studies (1503 patients) reported the rate of minimally invasive surgeries after neoadjuvant immunotherapy, including 3 RCTs, 8 non-randomized controlled studies, and 24 single-arm studies (**sTable 1**). The percentage of minimally invasive surgeries among all the studies ranged from 2.86% to 100.0%, and there were no correlations between minimally invasive surgery with disease stage or neoadjuvant regimen.

### Short-term clinical outcomes

Neoadjuvant immunotherapy alone can help decrease tumor stage and may even completely eradicate tumor mass, and subsequent surgery allows evaluation of its short-term benefit. Therefore, we systematically reviewed the short-term outcomes (pCR and MPR) of neoadjuvant immunotherapy reported in all the published studies (**sTable 2**).

#### Pathologic complete response

A pCR, defined by the disappearance of malignant residual tumor based on pathological detection, is a critical indicator of the complete removal of a malignancy (30). 57 studies (1842 patients) evaluated pCR after neoadjuvant immunotherapy, including 5 RCTs, 10 non-randomized controlled studies, and 42 single-arm studies (**Figure 2E**). A single-arm study of 41 patients who received nivolumab combined with chemotherapy as a neoadjuvant treatment reported 63.41% of these patients achieved pCR. 42 studies evaluated pCR in response to immunotherapy combined with chemotherapy as neoadjuvant treatment, and most of them achieved pCR in the range of 30 to 40%. Fourteen studies examined mono immunotherapy as neoadjuvant treatment, and 8 of them had pCR exceeding 10%, and the highest pCR was 48.0%. Only a few studies, all with small sample sizes, examined two immunotherapy agents (anti-PD-1/PD-L1 and anti-CTLA-4) or immunotherapy combined with radiotherapy as neoadjuvant treatment. Studies using two ICIs as neoadjuvant therapy reported higher pCR (33.3% and 37.5%) than a single agent (**Figure 2E**).

#### Major pathologic response

MPR, defined by 10% or less of residual viable tumor after preoperative therapy, is a valid surrogate endpoint for survival and provides a rapid means for comparing different neoadjuvant regimens. 56 studies (1841 patients) reported MPR rates, including 5 RCTs, 12 non-randomized controlled studies, and 39 single-arm studies (**Figure 2F**). Most evidence (11 studies) examined patients with clinical stages I to III. 14 of the 56 studies used mono-immunotherapy as neoadjuvant therapy, two used a combination of dual ICIs, and 43 used combination therapies of ICI with chemotherapy or radiotherapy. None of these 56 studies exclusively examined patients with clinical stages I to II. The MPR varied from 12.5% (a RCT of a single anti-PD-1/PD-L1 agent as neoadjuvant therapy) to 100.0% (a non-randomized controlled studies of an anti-PD-1/PD-L1 plus chemotherapy neoadjuvant therapy). Studies that examined NSCLC patients with more advanced NSCLC tended to achieve higher MPR rates.

### Long-term clinical outcomes

Clinical studies of the long-term clinical outcome of perioperative immunotherapy usually report survival parameters, such as OS, DFS, EFS, PFS, and RFS. However, these studies differed in their presentations of survival data, and only a limited number of studies provided specific survival data, so evidence mapping was unsuitable. Instead, we summarized the details of treatment regimens, including drug, dosage, treatment duration, and corresponding outcome parameters, using a tabular review (**sTables 3**–**7**).

### Overall survival

Only a few studies reported data regarding OS, mainly because most of the studies examined here began very recently (**sTable 3**). 21 studies (2110 patients) reported OS data after perioperative immunotherapy and surgery, including 9 that examined single-agent immunotherapy as neoadjuvant treatment, 12 that examined immunotherapy combined with chemotherapy as neoadjuvant treatment. The survival rate was similar among these studies, and the lowest OS at 12-months (82.2%) and 18-months (73.0%) occurred when chemotherapy was combined with anti-CTLA-4 as neoadjuvant treatment. Of all studies that reported OS, only 1 study reported achieving a median OS; this study examined chemotherapy combined with anti-CTLA-4 as neoadjuvant treatment and reported a median OS of 29.2 months. 14 studies (1797 patients) did not reach median OS.

#### Disease-free survival

Fifteen studies (1932 patients) reported DFS, including 3 RCTs, 4 non-randomized controlled studies and 8 single-arm studies (**sTable 4**). Eleven studies reported median DFS, with a median range of 17.9-53.6 months, of which seven studies did not achieve median DFS.

#### Progression-free survival

Five studies (155 patients) reported PFS, although none achieved a median PFS (31) (**sTable 5**). One study reported a 6-month PFS of 55.60%, a 1-year PFS of 99.65%, and one reported a 2-year PFS of 88.19%. The discrepancy among these studies might be attributed to their use of different definitions of PFS. Additionally, three studies (78 patients) reported median PFS, One study achieved median PFS of 11.3 months, and the median PFS was not reached in another 2 studies.

#### Relapse-free survival and event-free survival

Five studies (263 patients) reported RFS data, and neither reached median RFS in 2 studies. All of these studies had reported 12-month, 18-month, or 24-month RFS, which are 100.0%, 73.00-79.70%, 69.00-93.30%, respectively (**sTable 6**). Only 3 studies reported EFS, and its 12-month EFS was 73.40%, 36-month EFS was 70%. Median RFS ranged from 31.6 to 44.4 months in 2 studies, and the median PFS was not reached in another study. (**sTable 7**).

### Safety profiles

Only a few studies reported the safety profiles of perioperative immunotherapies (**sTable 8**). In particular, 24 studies (1914 patients) reported the incidence of treatment-related adverse events (TRAEs) or adverse events (AEs) of any grade, including 9 RCTs and 15 single-arm studies. In general, relative to studies that examined a single immunotherapy agent as neoadjuvant therapy, the incidence of TRAEs and AEs was higher in studies that combined an ICI with chemotherapy or used dual ICIs as neoadjuvant therapy. For example, one single-arm study examined a CTLA-4 inhibitor plus chemotherapy as neoadjuvant therapy and CTLA-4 inhibitor monotherapy as adjuvant therapy and reported that 1 of 24 participants did not receive surgery due to AEs.

Sixteen studies (1582 patients) reported data regarding severe AEs (sAEs), including 8 RCTs, 1 non-randomized controlled study, and 7 single-arm studies (**sTable 8**). The highest incidence of sAEs (88.06%) was in the study with 67 patients that examined the combination of immunotherapy (anti-PD-L1) with chemotherapy as neoadjuvant treatment plus adjuvant treatment with immunotherapy (anti-PD-L1) ± radiotherapy. A high incidence of sAEs also occurred in a study that examined the combination of immunotherapy (anti-PD-1) with chemotherapy as neoadjuvant therapy (40.91%), and another study that examined immunotherapy (anti-PD-1) as neoadjuvant therapy (34.62%). The incidences of sAEs in all other studies were below 30%. Studies that examined a single ICI as a neoadjuvant treatment had a lower incidence of sAEs than those that examined more than one agent (two ICIs or an ICI combined with chemotherapy; **sTable 4**). Eight studies (4 RCTs, 1 non-randomized controlled study and 3 single-arm studies) reported death-associated TRAEs, and the incidence ranged from 0.69% to 11.11%. Almost all deaths were attributed to immune-related interstitial pneumonia, except for one patient who died of postoperative acute respiratory distress syndrome (ARDS). No other studies reported deaths due to TRAEs.

## Discussion

The potential application of immunotherapy as a neoadjuvant or adjuvant treatment for NSCLC is a research hot topic. In addition, ICIs as perioperative treatments appear to provide impressive therapeutic benefits for patients with NSCLC. Many systematic reviews also examined this topic and identified the clinical significance of various therapeutic regimens that use ICIs for neoadjuvant or adjuvant treatment. However, none of these previous reviews used evidence mapping to summarize all relevant clinical trials, a method that provides simple visual displays of complex, diverse, and abundant information. Thus, we used evidence mapping and tabular review to systematically summarize immunotherapy as neoadjuvant and adjuvant treatment for NSCLC by reviewing previous clinical trials and examining study designs, study populations, therapeutic outcomes, and safety profiles. We hope that our study provides insight regarding the current clinical status of using immunotherapy as a perioperative treatment for NSCLC and that it also helps to identify gaps that should be addressed in future studies.

Among the 41 studies we examined, only 8 were RCTs; the others were single-arm clinical trials or NRCTs. This means that only a limited amount of high-quality evidence is available. Notably, most of these 41 studies examined the effect of ICIs as neoadjuvant treatments. This is likely because evaluating the outcomes of neoadjuvant therapies is typically more accessible and less time-consuming than evaluating the outcomes of adjuvant treatments. However, many trials are currently evaluating the therapeutic benefits of adjuvant ICIs (32–37), and we expect more clinical data will be published in the coming years.

The clinical benefit of perioperative ICIs for NSCLC was the primary focus of all 41 clinical trials that we examined. Among short-term clinical outcomes from neoadjuvant ICIs, post-surgical pCR and MPR are the most feasible and most used to assess clinical benefits. Our evidencing mapping results indicated that the rates of pCR (6.94–75%) and MPR (7.69–100%) varied dramatically among studies that used ICIs as neoadjuvant treatments. Previous studies have demonstrated the correlation between MPR and OS (38, 39). However, the definition of MPR is still controversial. The existing histological definition of MPR was ≤10% residual active tumor in the primary lesion of NSCLC and does not require examination of tumor residual in lymph nodes (40). However, it has also been suggested that MPR of lymph nodes was of great value in predicting prognosis for lung cancer, while MPR of lymph nodes with primary lesions has a higher predictive value for prognosis (41). In addition, it has been shown that the optimal threshold values for predicting survival in different histological types of tumors may differ, with 10% and 65% for squamous cell carcinoma and adenocarcinoma, respectively (42, 43). There are still differences in the judgment of different pathologists in determining residual active tumor ≤10% at the time of pathological examination. Therefore, the value of MPR as a surrogate indicator in neoadjuvant therapy still needs to be confirmed in more clinical studies. Many factors could contribute to these variations, including differences in sample sizes, neoadjuvant regimens, and tumor stages. However, our evidence mapping indicated some general trends. For example, combination regimens (ICI combined with chemotherapy or two ICIs) provided better short-term clinical outcomes than single-agent ICIs as neoadjuvant treatment, consistent with previous research that reported that immunotherapy combined with chemotherapy was more beneficial than chemotherapy, a single agent for advanced NSCLC (44). This is likely because immunotherapy and chemotherapy have different tumoricidal effects (45) and because chemotherapy can sensitize a tumor to immunotherapy due to its activation of the immune microenvironment (46). Other studies also indicated an additive anti-tumor effect when using a combination of an anti-PD-1/PD-L1 and anti-CTLA-4 immunotherapy (47). Although these treatments all elicit anti-tumor effects by activating the immune response, they have distinct mechanisms (48). Our pooled analysis of studies that used neoadjuvant ICIs also indicated that two ICIs provided more benefits than a single ICI.

Although it is more expedient to evaluate short-term outcomes for the rapid analysis of results, long-term parameters, such as OS, PFS, and DFS, are more clinically relevant for assessing the therapeutic benefits of treatments. However, data on these long-term outcomes were only available in a limited number of our studies. Therefore, we cannot infer any relationship between the perioperative immunotherapy regimens described here with long-term outcomes. In addition, although pCR is a widely applied indicator of clinical outcome in most clinical trials, only a few studies examined OS and other long-term indicators and none of the studies we examined assessed the relationship between pCR and OS in NSCLC patients. Thus, more studies are needed to evaluate the long-term outcomes of NSCLC patients who receive perioperative ICIs. For instance, the OS was defined as the primary outcome in the KEYNOTE-671 study (49).

In addition to the therapeutic benefits of perioperative immunotherapy regimens, we also examined their potential AEs. It is always important to consider the impact of neoadjuvant treatment on the subsequent surgery. Our analysis indicated that the rates of surgery and R0 resection were high (about 90%) among all clinical trials, and there were no variations associated with tumor stage, neoadjuvant regimen, or study design. These findings indicated that neoadjuvant immunotherapy did not interfere with the execution of surgery, regardless of neoadjuvant regimen or tumor stage. However, the rate of surgery delay varied from 0% to 20.81% among studies, although this could be partially attributed to the different definitions of “surgery delay” among the different trials. We also observed more significant surgery delay in RCTs than in single-arm studies, but there was no relationship between surgery delay and therapeutic regimen or tumor stage.

The safety profile of a therapeutic regimen is a critical issue. Our review indicated that perioperative ICIs, either single or multiple agents, were associated with varying degrees of AEs. The incidence of overall AEs varied from 3.33% to 100%, the incidence of sAEs varied from 0% to 45.83%, and the incidence of sAEs was higher in trials that examined anti-CTLA-4 immunotherapy, especially when it was combined with chemotherapy. This is consistent with previous studies which reported that anti-CTLA-4 immunotherapy was a more toxic treatment than anti-PD-1/PD-L1 immunotherapy (50, 51). Our review also indicated that combination treatment was associated with a higher incidence of AEs and more sAEs than a single ICI as perioperative treatment, consistent with using ICIs in advanced irresectable patients. In general, NSCLC patients had good tolerance of perioperative treatment using ICIs (both single use and combined with other therapies), and the incidence of death associated with AEs was at or close to 0% in all studies, regardless of perioperative regimen.

Among all examined studies, only a few assessed immunotherapies as adjuvant therapy. A well-known study examining atezolizumab as adjuvant immunotherapy after adjuvant chemotherapy for resected stage IB-IIIA NSCLC (IMpower010) showed pronounced benefit in the subgroup of patients whose tumors expressed tumors PD-L1 on 1% or more of tumor cells (52). In addition, the KEYNOTE-091 trial, a phase 3 triple-blinded randomized controlled trial, showed that pembrolizumab as adjuvant treatment for patients with stage IB to IIIA NSCLC had superior DFS with active therapy vs placebo (53). Based on the encouraging results of adjuvant immunotherapy on NSCLC, we can see the positive value of immunotherapy in reducing the risk of relapse in NSCLC patients. However, some issues still need to be figured out for optimal efficacy and safety of the treatment. First, the target patients should be defined. For instance, the expression level of PD-L1 was an independent predictor of the efficacy of adjuvant atezolizumab treatment in the IMpower010 study (52), but not in the KEYNOTE-091 (53). In addition, there are still many questions about adjuvant immunotherapy waiting for us to further answer through research data. Some related research questions are: Is PD-L1 a prognostic biomarker or immunotherapy predictive biomarker in early NSCLC? Are there opportunities for benefit from adjuvant immunotherapy in patients with actionable mutations? And are there new biomarkers that can better predict the use of adjuvant immunotherapy?

The impact of neoadjuvant immunotherapy on the subsequent execution of surgery is also a critical issue. Neoadjuvant treatment could promote tumor shrinkage and facilitate tumor resection, but it could also delay surgery and reduce the opportunity for R0 resection. Thus, evaluating the surgical outcome associated with different neoadjuvant immunotherapy regimens is essential. Although our study showed no significant difference in the rates of surgery, surgery delay, and R0 resection among studies with different regimens or study designs, we suggest further examination of surgical outcomes using head-to-head analyses.

Our pooled visual analysis of all studies that used ICIs as perioperative treatments for NSCLC increased the understanding of this topic and helped identify evidence gaps that should be addressed in further studies. One evidence gap is that most studies on this topic investigated patients with stage III NSCLC. Evidence regarding the therapeutic benefit and safety profiles of ICIs as perioperative treatments for early-stage NSCLC (stage I-II) is limited. A second evidence gap is that only a small fraction of NSCLC patients experiences impressive responses to ICI treatments, even though it is a milestone treatment for some patients. In particular, numerous biomarkers, such as PD-1/PD-L1, T cell infiltration, TMB, and others, are helpful for the selection of non-operable patients who benefit most from immunotherapy (54–56). However, studies that examined ICIs as perioperative treatments usually do not use biomarkers for patient selection. PD-1/PD-L1 expression status is the most widely used biomarker in selecting patients for non-perioperative immunotherapy (57). It has not yet been determined whether it is necessary to consider PD-1/PD-L1 expression to apply ICIs as perioperative treatments. When using neoadjuvant immunotherapy, determining PD-L1 expression status and other biomarkers is more challenging and less feasible. Alterative biomarkers, such as imaging data, liquid biopsies, and even some clinical characteristics, could have the potential to predict the efficacy of neoadjuvant immunotherapy (58, 59), a topic that needs to be examined in the future. An ongoing clinical trial (MERMAID) is examining durvalumab as adjuvant therapy for NSCLC to determine the optimal timing and suitable biomarkers for adjuvant immunotherapy, and we eagerly anticipate the results. A third evidence gap is that very few studies have examined the combination of ICIs with other treatments, such as radiotherapy, antiangiogenic therapy, and targeted therapy, as perioperative treatments for NSCLC. Further studies are also needed on this topic.

Our review is the first to use evidence mapping to examine ICIs as perioperative treatments for NSCLC, which can contribute for the exploration of perioperative immunotherapy. In future research, perioperative immunotherapy, including short-term and long-term benefits, should be improved by exploring immune-based treatment models, including the combination of drugs and the timing and duration of drug use. In addition, the target patients should be defined precisely through the improvement and exploration of detection technology. Nonetheless, some limitations need to be addressed. First, this review systematically sought to summarize all currently available data on this topic, but a pooled statistical analysis was not possible because of the diversity of the included data. Thus, any conclusions derived from our study are observational and currently lack statistical support. Second, due to the limitations of available data and the difficulty of performing pooled analysis, we only analyzed some data using a tabular format rather than evidence mapping. Finally, because the rapid development in the field and the emerging new research are limited to our literature search time, the research data of the published research results after that time node were not included in this analysis.

## Conclusion

This review systematically and efficiently summarized the therapeutic effects and safety profiles of ICIs as perioperative treatments for NSCLC. Our study provided an in-depth understanding of this topic and helped to identify evidence gaps that need to be addressed in further studies.

## Data availability statement

The raw data supporting the conclusions of this article will be made available by the authors, without undue reservation.

## Author contributions

YN, JL, WH and JW contributed equally. Guarantor for the overall content: TJ. Study concept and design: YN, TJ, JL, WH and JW. Drafting of the manuscript: YN and KL. Statistical analysis: HG, FL and SK. Study supervision and organisation of the project: TJ. Technical consultant: WH. All authors contributed to the article and approved the submitted version.
